# Low Profile Triangle-Shaped Piezoelectric Rotary Motor

**DOI:** 10.3390/mi15010132

**Published:** 2024-01-13

**Authors:** Andrius Čeponis, Vytautas Jūrėnas, Dalius Mažeika

**Affiliations:** 1 Laboratory of Robotics and Piezomechanics, Institute of Mechatronics, Kaunas University of Technology, Studentų Str., 56, 44249 Kaunas, Lithuania; vytautas.jurenas@ktu.lt; 2 Department of Information Systems, Faculty of Fundamental Sciences, Vilnius Gediminas Technical University, Saulėtekio Avn., 11, 10223 Vilnius, Lithuania; dalius.mazeika@vilniustech.lt

**Keywords:** piezoelectric motor, angular motion, inertial operation principle, low-profile motor

## Abstract

In this paper, we present research on a novel low-profile piezoelectric rotary motor with a triangle-shaped stator. The stator of the motor comprises three interconnected piezoelectric bimorph plates forming an equilateral triangle. Bimorph plates consist of a passive layer fabricated from stainless steel and four piezo ceramic plates glued to the upper and lower surfaces. Furthermore, spherical contacts are positioned on each bimorph plate at an offset from the plate’s center. Vibrations from the stator are induced by a single sawtooth-type electric signal while the frequency of the excitation signal is close to the resonant frequency of the second out-of-plane bending mode of the bimorph plate. The offset of the spherical contacts allows for a half-elliptical motion trajectory. By contrast, the forward and backward motion velocities of the contacts differ due to the asymmetrical excitation signal. The inertial principle of the motor and the angular motion of the rotor were obtained. Numerical and experimental investigations showed that the motor operates at a frequency of 21.18 kHz and achieves a maximum angular speed of 118 RPM at a voltage of 200 V_p-p_. Additionally, an output torque of 18.3 mN·mm was obtained under the same voltage. The ratio between motor torque and weight is 36 mN·mm/g, while the ratio of angular speed and weight is 28.09 RPM/g.

## 1. Introduction

Contemporary equipment and systems dedicated to research, manufacturing, visualization, and free space communication rely on high-precision mechatronic motion and manipulation devices and their components. These systems control the position and orientation of optical devices by focusing, pointing, and intensely modulating devices associated with laser beams [1,2,3]. Therefore, mechatronic systems must provide high-resolution motion, good repeatability, and optimal performance. Furthermore, in response to contemporary demands, these systems and devices must meet weight, physical footprint, and versatility criteria in both operation and installation.

However, in most cases, mechatronic systems are manufactured from electromagnetic motors and actuators [4,5,6]. Incorporating electromagnetic drives into high-precision mechatronic motion systems presents challenges, such as electromagnetic interference with other electronic devices, limited scalability, and the need for additional motion transfer mechanisms. Furthermore, despite these constraints, electromagnetic actuators and motors also face limitations in motion resolution, which include mass, size, and footprint minimization [7,8]. To mitigate these limitations, it becomes essential to explore alternatives to electromagnetic drives. Piezoelectric actuators and motors are possible options [9,10,11]. These types of drives provide high motion resolution, demonstrate good scaling and dynamic characteristics, and are magnetic field-free. Moreover, they can be engineered to be lightweight, compact, and space-efficient, enabling rotor driving without additional motion transfer systems [12,13,14]. Piezoelectric drives can drive the payload linearly and angularly using the same actuator. However, the designs and excitation schematics of these drives are more complex than those of purely angular or linear piezoelectric drives [15,16,17]. Considering these state-of-the-art drives, there is a significant demand for actuators with simple design, excitation, and mounting schemes that can be directly mounted onto printed circuit boards (PCB). 

Huang et al. described a small inertial piezoelectric rotary motor [18]. The motor was based on a piezoelectric shear stack and a disc-shaped rotor preloaded via a friction block. The stack is driven by a single sawtooth-type electric signal that generates shear-type deformations of the block, which are used to induce the rotor’s angular motion under the inertial principle. The authors reported that the proposed motor could provide up to 5.86 µrad/s of angular motion speed while motion resolution reaches up to 0.64 µrad. 

Wang et al. introduced a piezoelectric inertial rotary motor comprising two piezoelectric multi-layer actuators positioned within a rhombic mechanism complemented by a ball-type bearing used as a rotor [19]. The multi-layer actuators are fixed at one end while the other end is preloaded onto the ball-bearing rotor using springs. Actuators are placed on opposite sides of the rotor. The motor excitation is achieved through the synchronized application of a sawtooth signal to both actuators simultaneously. Such excitation ensures that the actuators expand and contract at different velocities and rotor rotation is achieved under the inertial operation principle. Numerical and experimental investigations showed that the proposed motor can achieve an angular resolution of 3.5 μrad while the maximum angular velocity reaches 0.44 rad/s. 

Chiang et al. introduced a small-size piezoelectric rotary motor consisting of a hollowed square-shaped tube and four piezoelectric plates [20]. The two-sided rotor is placed on the tube’s upper and lower surfaces and is preloaded by a spring. The size of the motor is 1.6 × 1.6 × 2 mm and the weight is 25 mg. Motor operation is based on the excitation of traveling wave vibrations employing the tube’s two perpendicular bending modes while four harmonic signals with a phase difference of π/2 are applied. Numerical and experimental investigations were performed and showed that the proposed motor can achieve an angular speed of 1800 rad/s. 

State-of-the-art piezoelectric motors can solve complex motion or positioning problems in different technological and scientific fields via high motion speed and resolution as well as compact size. Moreover, the advantages of piezoelectric motors, such as magnetic field-free operation, gear-free direct drive of loads, and self-locking abilities make these motors highly attractive compared to electromagnetic or other types of motion sources [21,22]. On the other hand, complex driving schematics, high operation frequencies, as well as these motors’ lack of direct integration abilities with small devices limit their application [23]. This paper presents numerical and experimental investigations of a novel low-profile piezoelectric motor that can be directly mounted to a printed circuit board (PCB). It allows flexible integration and application options for the proposed motor design. Moreover, the motor has a good scalability feature because of the low-order vibration mode used for its operation. It noticeably reduces the total size of the motor, while its operation frequency remains relatively low. Finally, the motor is driven by a single sawtooth-type signal, which simplifies motor excitation schematics. 

The paper is organized as follows: Section 2 describes the design and operating principle of the motor; Section 3 presents the results of numerical modeling and simulation; Section 4 describes the prototype, a description of the experimental setup, and measurement results. Finally, Section 5 provides our conclusions.

## 2. Design and Operation Principle of the Motor

**Figure 1:**
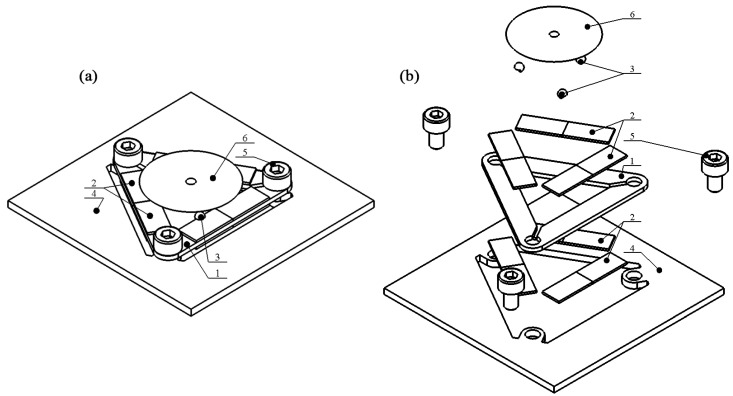
Design of motor; (**a**)—assembled view of motor; (**b**)—exploded view of motor; 1—triangle-shaped stator; 2—piezoceramic plates; 3—spherical contact; 4—printed circuit board; 5—bolts; 6—cone-shaped rotor.

**Figure 2:**
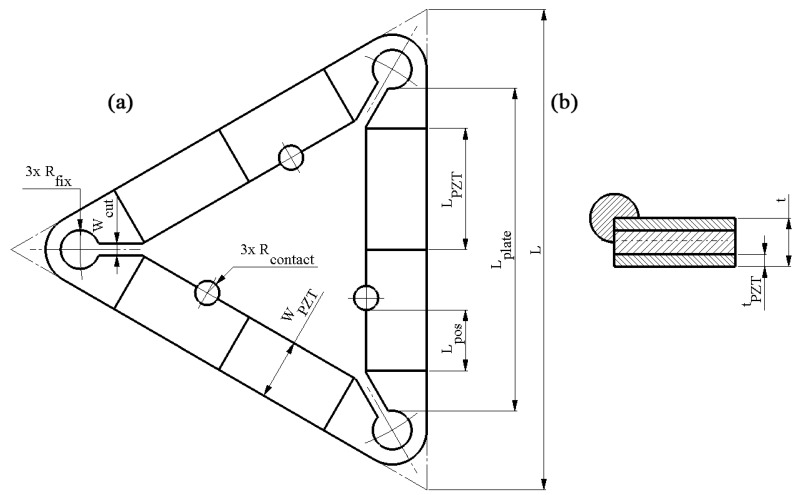
Schematics of the stator; (**a**)—top view; (**b**)—section view.

**Figure 3:**
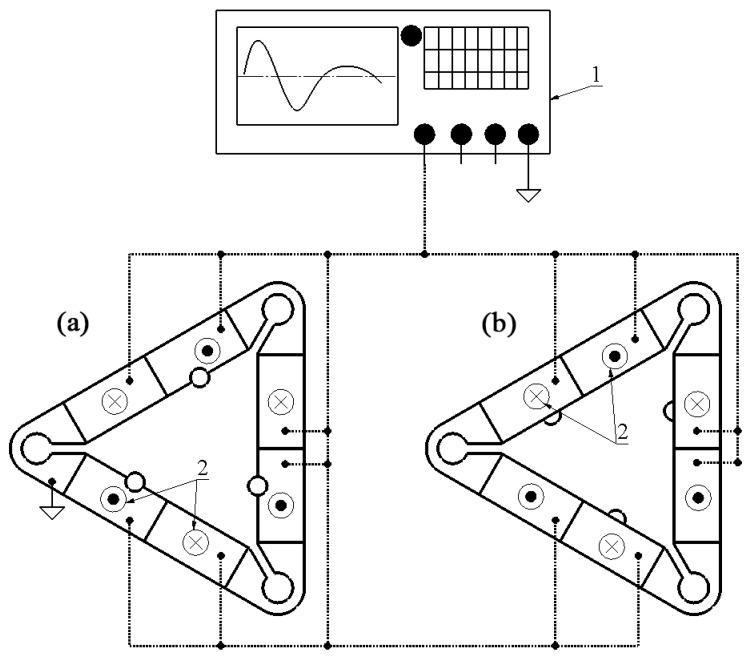
Excitation schematics of motor; (**a**)—top view; (**b**)—bottom view; 1—signal generator; 2—polarization direction.

The proposed piezoelectric motor has a simple and scalable design consisting of three interconnected piezoelectric bimorph plates that form a triangular structure. Each bimorph plate contains a passive layer fabricated from steel and four piezo ceramic plates glued onto the passive layer’s top and bottom surfaces (Figure 1 and Figure 2). The polarization direction of the piezoceramic plates is aligned with their thickness and oriented in opposite directions from the piezoceramic plates located on the same surface of the bimorph plate. Piezoceramic plates located on the passive layer’s top and bottom surfaces have the same polarization direction (Figure 3). The fully assembled triangle-shaped stator comprises a total of twelve piezoceramic plates. Furthermore, each piezoelectric bimorph plate features a spherical contact positioned along the inner edge of the plate with an offset from the plate’s center. The stator is fastened to a printed circuit board (PCB) using three bolts positioned at the corners of the triangular-shaped stator. Finally, a cone-shaped rotor is positioned on top of the spherical contacts with a preload. The design of the motor is shown in Figure 1. The schematics of the stator and its geometrical characteristics are shown in Figure 2 and Table 1, respectively. The values of geometrical parameters were obtained during the optimization study described in Section 3.

It must be noted that the proposed motor has a low profile. The total height of the stator is 2 mm while the standard printed circuit board (PCB) typically has a thickness between 1.6 and 2 mm. Furthermore, the motor’s footprint is 415 mm^2^ while the volume and mass without a rotor is 4519.49 mm^3^ and 4.2 g, respectively. Hence, the proposed motor is suitable for applications with stringent requirements on mass, footprint, and profile height.

Motor operation is based on the inertial principle, which is achieved through simultaneous excitation of the second out-of-plane bending modes of all three piezoelectric bimorph plates. A single periodic sawtooth-shaped electric signal with a frequency close to the second out-of-plane bending mode is used to excite the motor and is applied uniformly across all electrodes of the bimorph plates (Figure 3). This approach ensures the generation of asymmetric out-of-plane vibrations within the stator.

**Figure 4:**
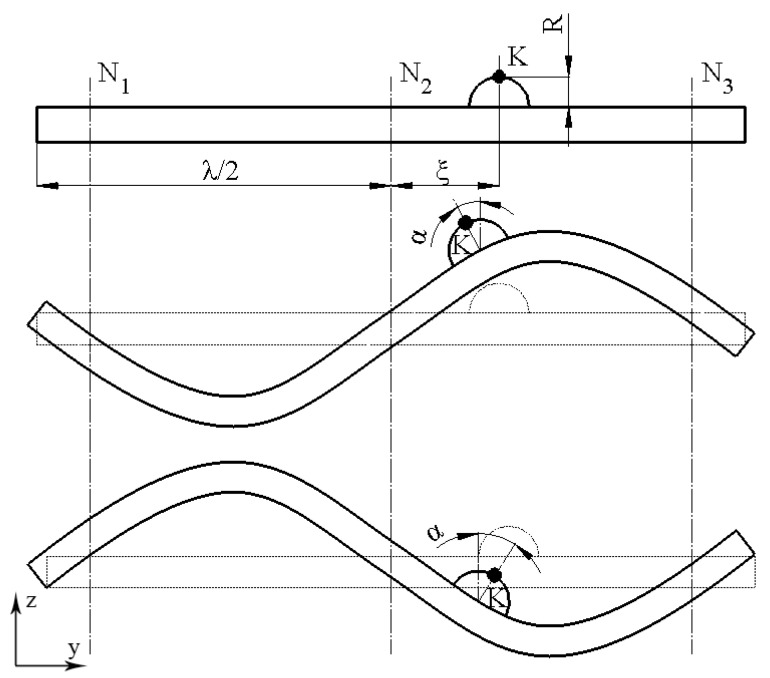
Positions of bimorph plates during motor operation; *K*—contact point; *R*—radius of spherical contact; N_1_, N_2_, and N_3_—nodal points in the second out-of-plane bending mode; *ξ*—offset of spherical contact; *λ*—wavelength; *α*—inclination angle of contact point inclination.

To obtain the angular motion of the conical rotor, spherical contacts are placed in an offset position from the center of bimorph plates, close to the antinode of the bending vibrations. Such a position ensures that during excitation of the second out-of-plane vibration mode, the contact generates a curved motion trajectory in the plane. Following this trajectory, the contact impacts the rotor, causing it to start rotating via the stick–slip principle. Figure 3 provides the motor’s excitation schematics while Figure 4 defines motor operation. 

Considering Figure 4, the position of contact point *K* on the bimorph plate can be described as follows:

(1) $$y_{K}\left(\xi \right)=\frac{\lambda }{2}+\xi ;$$

where, *y_K_* is the horizontal position of the contact point *K* on the bimorph plate; *ξ* is the offset of the contact point *K* from the nodal point N_2_ of the second bending mode; *λ* is the wavelength. Therefore, bending vibrations of the plate are induced, and the vertical displacement of contact point *K* can be described as follows:

(2) $$z\left(y_{K}\right)=A\sin\left(\frac{2\pi }{\lambda }y_{K}\right);$$

where *A* is the plate vibration amplitude of the bending vibrations and *λ* is the wavelength. Plate vibration amplitude *A* depends on the excitation signal amplitude, the piezoelectric constant, and the geometric characteristics of the plate such as the length and thickness of the passive and piezoelectric layers, as well as their material characteristics [24]. Furthermore, the plate deforms the spherical contact and is rotated by an angle of *α*, which induces the motion of the contact point *K* in the horizontal direction:

(3) $${∆y}_{K}=Rsin\left(\alpha \right)$$

where *R* is the radius of the spherical contact and α is the angle of the spherical contact. Based on the geometrical relations, the displacement of the *K* point in the vertical direction can be described as follows:

(4) $$∆z_{K}=Asin\left(\frac{2\pi }{\lambda }y_{K}\right)-R\left(1-cos\left(\alpha \right)\right)$$

Angle *α* can be expressed as follows:

(5) $$\alpha \approx \frac{\partial z_{k}}{\partial y}=A\frac{2\pi }{\lambda }cos\left(\frac{2\pi }{\lambda }y_{k}\right)$$

Considering that the vibration amplitude *A* and angle *α* are small, it can be assumed that sin (*α*) ≈ *α* and cos (*α*) ≈ *1*. Therefore, the displacement of contact point *K* can be described as follows:

(6) $$\left\{\begin{array}{c} ∆y_{K}=RA\frac{2\pi }{\lambda }\;cos\left(\frac{2\pi }{\lambda }y_{K}\right)\\ ∆z_{K}=A\sin\left(\frac{2\pi }{\lambda }y_{K}\right) \end{array}\right.$$

Therefore, considering that the plate vibrates at the bending with a wavelength of *λ* and *L* = *λ* in our case, then the total displacement amplitude of contact point *K* can be described as follows:

(7) $$u_{K}=\sqrt{A^{2}\left({cos}^{2}\left(\frac{2\pi }{L}\left(\frac{L}{2}+\xi \right)\right)\left(\frac{4\pi^{2}}{L^{2}}R^{2}-1\right)-1\right)}$$

The displacement amplitudes of contact point *K* in the Y direction are directly related to the radius of the spherical contact and the amplitudes of plate vibrations. The offset *ξ* of the spherical contact from the nodal point N_2_ plays an essential role. Therefore, the offset value must be optimized to obtain a proper ratio between the displacement amplitudes in the Y (*u_y_*) and Z (*u_z_*) directions. 

## 3. Numerical Investigation of Motor

A numerical investigation was performed to confirm the operation principle of the motor and indicate its mechanical and electromechanical characteristics. For this purpose, a numerical model of the motor was built using Comsol Multiphysics software (COMSOL, Inc., Burlington, VT, USA) using the geometrical parameters shown in Table 1. The following boundary conditions were applied, i.e., the motor was rigidly clamped in the fixing cavities while the electrical boundary conditions were established, as shown in Figure 3. Finally, material characteristics were included in the model. The material of the stator was DIN 1.4301 stainless steel, NCE81 (CTS Corp, Lisle, IL, USA) piezoelectric ceramics, and aluminum oxide. The glue layer was neglected in the model. Table 2 presents the material properties used to build a numerical model.

Firstly, a modal analysis of the motor was performed to determine a vibration mode suitable for motor operation. 

**Figure 5:**
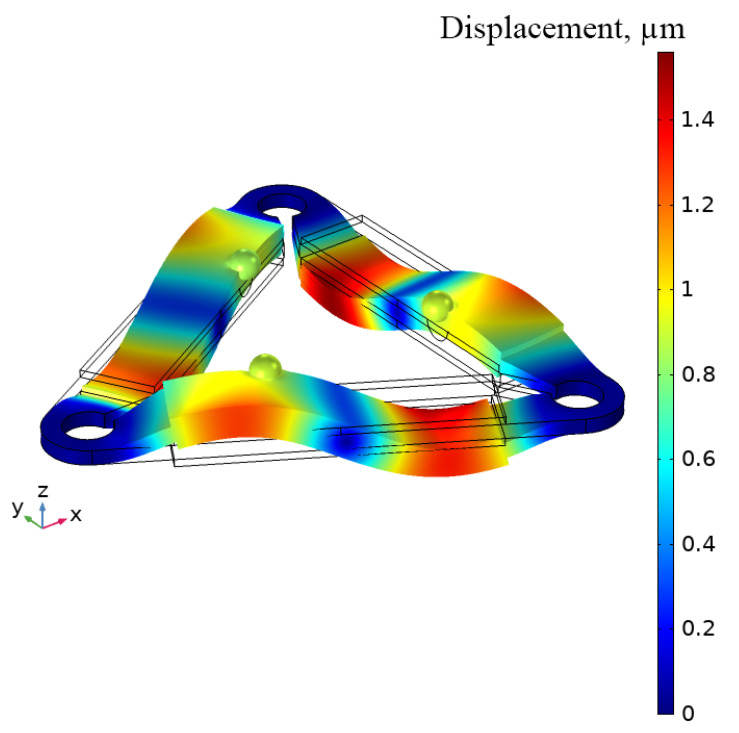
The modal shape of the motor at 22.27 kHz.

The second out-of-plane bending mode of piezoelectric bimorph plates was obtained at the frequency of 22.27 kHz (Figure 5). Furthermore, spherical contacts have motions in two directions, i.e., vertical and horizontal. Our results confirm the assumption that two directions of contact motion are caused by the asymmetric position of spherical contacts.

**Figure 6:**
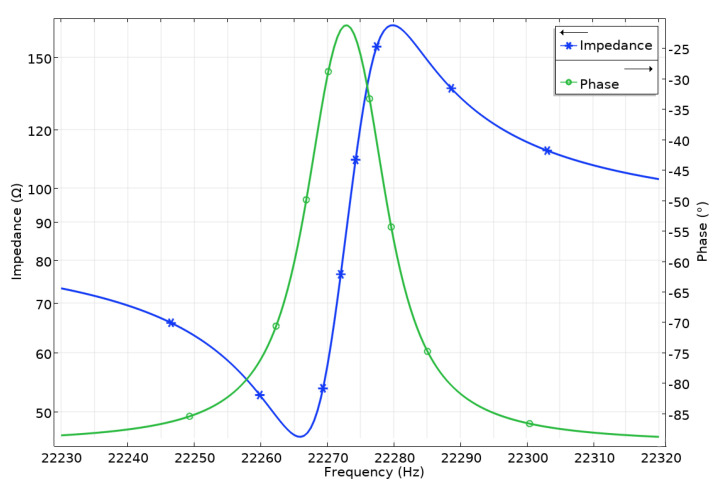
Impedance and phase frequency characteristics of the motor.

The next step of the numerical investigation was dedicated to calculating the motor’s impedance and phase frequency characteristics. For this purpose, the frequency domain study was set up in the range of 22.23–22.32 kHz with a step of 1 Hz. Figure 6 shows the calculation results.

The resonant frequency of the second out-of-plane bending mode was 22.266 kHz. The difference between the resonant and natural frequencies does not exceed 1%. This mismatch occurred due to differences in the calculation steps, i.e., the frequency domain study used a discrete frequency step of 1 Hz. Different electrical boundary conditions are used in the models, resulting in a minor difference. Furthermore, the electromechanical coupling coefficient of the motor *k_eff_* was calculated to be 0.049.

The next step of our numerical investigation was to find the optimal position of the spherical contact on the piezoelectric bimorph plate. For this purpose, an optimization study in the frequency domain was set up. The objective of our study was to maximize the ratio between displacement amplitudes in the Z (*u_z_*) and Y (*u_y_*) directions of contact point vibrations. The mathematical description of the optimization problem is shown below.

(8) $$\underset{L_{\mathrm{pos}}}{\max}\left(\frac{u_{z}}{u_{y}}\left(L_{pos}\right)\right)$$

subjected to

(9) $$L_{pos}^{min}\leq L_{pos}\leq L_{pos}^{max}$$

where, *L_pos_* is the horizontal position of spherical contact on the plate; $L_{pos}^{min}$ is the lowest value of spherical contact position; $L_{pos}^{max}$ is the highest value of spherical contact position; *u_z_* is the displacement amplitude of spherical contact in the Z direction; *u_y_* is the displacement amplitude of spherical contact in the Y direction. 

**Figure 7:**
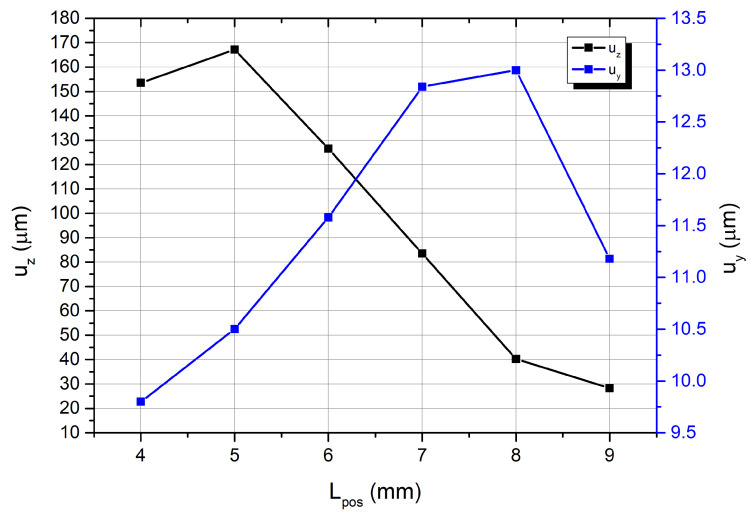
The amplitudes of spherical contact displacement in the Z and Y directions.

Constraints $L_{pos}^{max}$ and $L_{pos}^{min}$ were set to 4 and 9 mm, respectively, during calculation. The increment step was set to 1 mm, and the excitation signal amplitude was set to 100 V_p-p_. Figure 7 shows the calculation results. 

**Figure 8:**
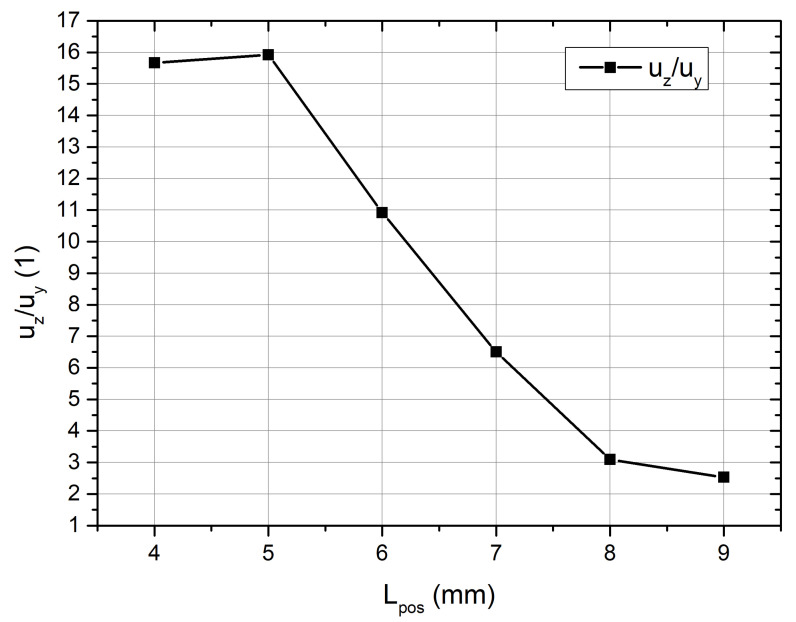
Ratios between displacement amplitudes in the Z and Y directions.

Amplitudes of spherical contact displacement in the Z direction range from 167.2 µm at a *L_pos_* of 5 mm to 28.3 µm at a *L_pos_* of 9 mm, whereas displacements in the Y direction vary from 9.8 in *L_pos_* of 4 mm µm to 13.1 µm at *L_pos_* of 8 mm. Therefore, the position of the spherical contact is essential for proper motor operation. To indicate the best position of spherical contact, the ratio between displacement amplitudes in the Z and Y directions was calculated (Figure 8). The aforementioned ratio varied, ranging from 2.53 times at a *L_pos_* of 9 mm to 15.92 times at a *L_pos_* of 5 mm. Hence, the highest ratio between displacement amplitudes occurred at a position 5 mm from the center of the bimorph plate. To study the motor experimentally, a prototype was constructed using this position.

**Figure 9:**
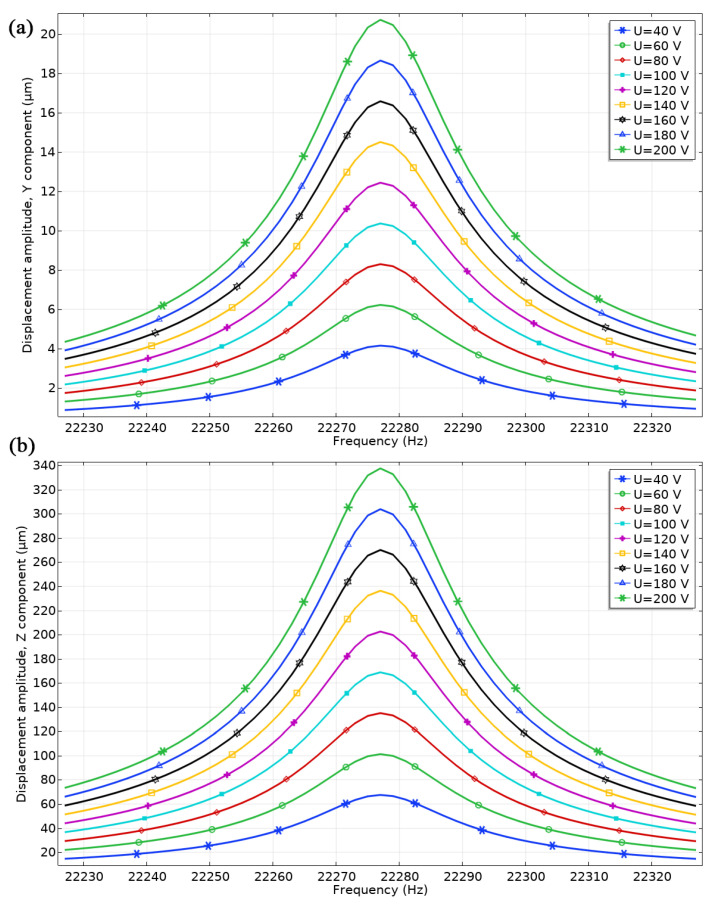
Displacement amplitudes at different excitation signal amplitudes; (**a**)—displacement amplitudes in the Y direction; (**b**)—displacement amplitudes in the Z direction.

A subsequent step in the numerical simulation involved calculating the dependence of displacement amplitudes in the spherical contact of the Z and Y directions based on excitation voltage. For this purpose, the frequency domain study was performed in a range from 23.04 to 23.22 kHz with a step of 1 Hz. The voltage was changed in the range of 40–200 V_p-p_ with a step of 20 V_p-p_. The results are shown in Figure 9. The highest displacement amplitudes were obtained at a frequency of 22.77 kHz. Displacement amplitudes in the Y direction varied from 3.18 to 20.72 µm or 0.16 to 0.1 µm/V_p-p_ when an excitation voltage of 40 or 200 V_p-p_ was applied, respectively. Displacement amplitudes in the Z direction reached 67.5 µm at a voltage of 40 V_p-p_ while a maximum amplitude value of 337.8 µm was obtained at 200 V_p-p_ or 3.37 and 1.69 µm/V_p-p_, respectively. Displacement amplitudes in the Z direction were more than 16 times higher than displacements in the Y axis when a voltage of 200 V_p-p_ was applied. Nevertheless, even though the displacement amplitudes in the Y direction are lower than those in the Z direction, the presence of displacement in the Y direction is essential as it guarantees the required motion trajectory for the motor to function properly.

**Figure 10:**
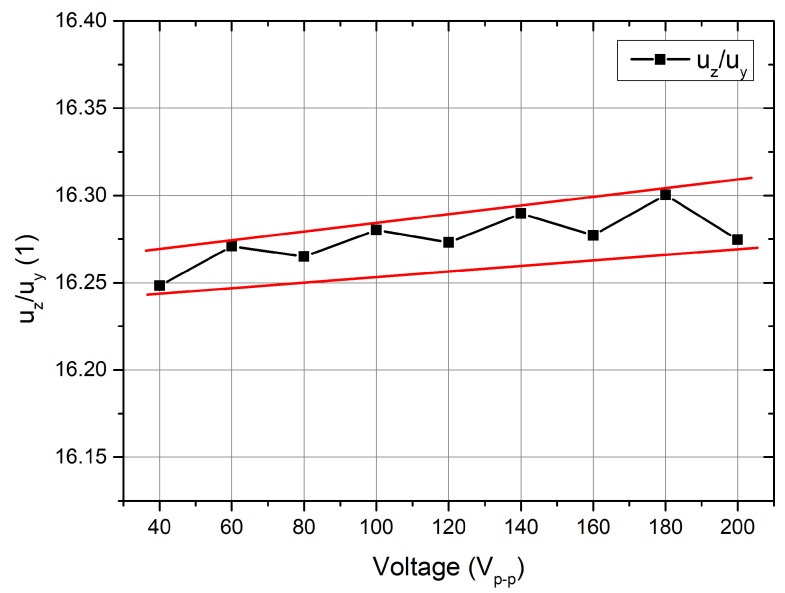
Ratios between displacement amplitudes at different excitation voltage amplitudes.

Furthermore, the ratios between displacement amplitudes in the Y and Z directions ($\frac{u_{z}}{u_{y}}$) were calculated at different excitation voltages (Figure 10). The ratio ($\frac{u_{z}}{u_{y}}$) slightly increases when the voltage increases. The average ratio value is 16.27, while the deviation does not exceed 0.054. These results show that the operation of the motor and its dynamic characteristics are linear and stable in the analyzed excitation voltage range. Moreover, the motor’s characteristics of the motor are predictable with controllable and stable operation under different conditions.

**Figure 11:**
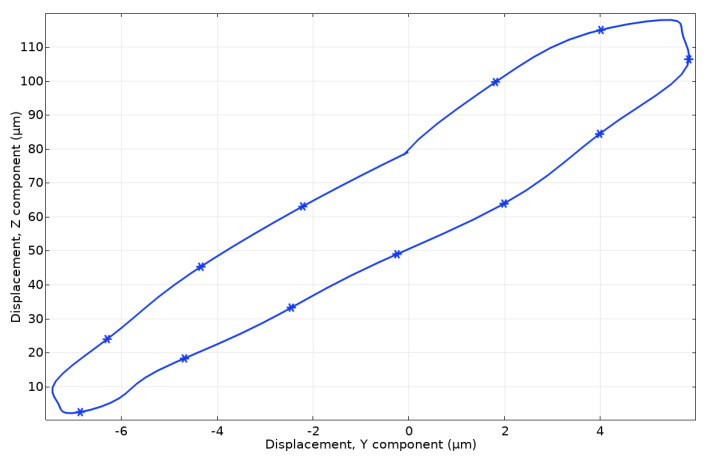
Motion trajectory of spherical contact.

The time-domain study was set up to indicate the motion trajectory of spherical contact (Figure 11). The study was established to calculate vibrations over one period of vibrations at a frequency of 22.266 kHz, as was indicated by impedance phase frequency characteristics. The excitation voltage was set to 100 V_p-p_ while the other boundary conditions were set as in previous studies.

The trajectory of spherical contact has a half-elliptical shape confirming the assumption that the asymmetric position of contact ensures motion in the Z and Y directions. The major axis of the trajectory lies on the Z axis and reaches around 140 µm, whereas the minor axis lies on the Y axis of motion and reaches around 14 µm. Thus, the calculated motion trajectory confirms the operation principle of the motor and shows that the asymmetric position of the spherical contact ensures the motor’s proper operation. 

**Figure 12:**
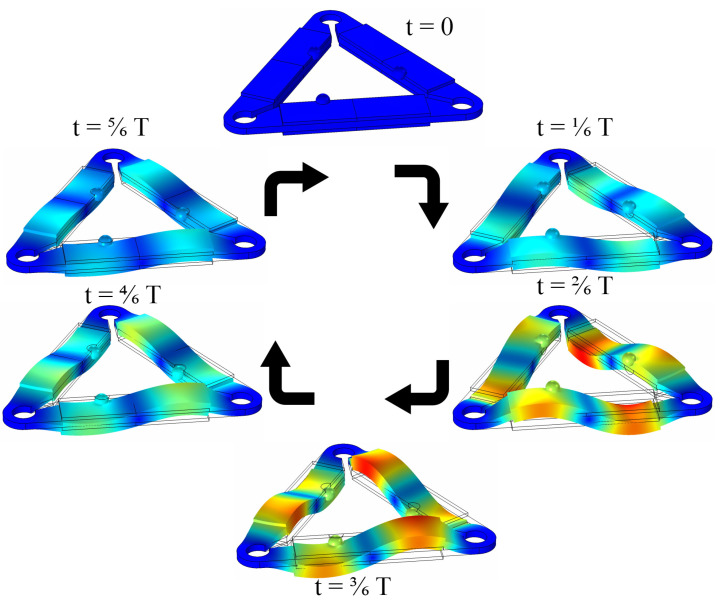
The operation sequence of the motor.

Finally, the operation sequence of the motor was simulated in the time domain. The time range of the simulation was set to one period T while the excitation signal amplitude and other boundary conditions were set as in the previous case. Figure 12 shows the simulation results.

As shown in Figure 12, simulations of the operation sequence confirmed the motor operation principle. Moreover, the inclination of the spherical contact was obtained during the operation, which confirmed the results of the contact point motion trajectory. 

The numerical investigations showed that the proposed motor design can ensure the angular motion of a cone-shaped rotor through the inertial stick–slip operation principle. Moreover, we found that the motor exhibits stable and predictable mechanical and electromechanical characteristics, which can be used to drive the motor in an open-loop control system. 

## 4. Experimental Investigations of Motor

**Figure 13:**
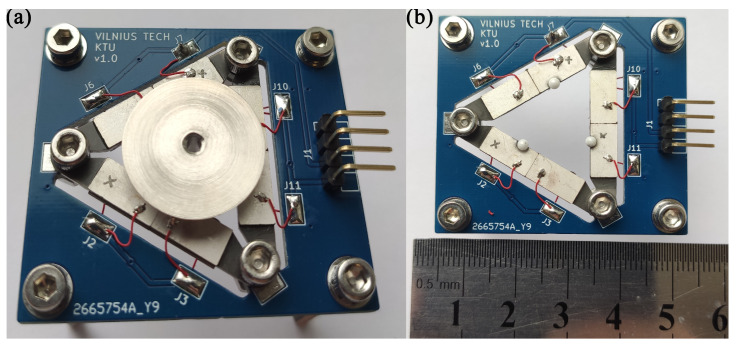
Prototype of motor; (**a**)—side view; (**b**)—top view.

The prototype of the motor was designed to perform experimental investigations (Figure 13). The motor was manufactured to strictly respect the geometrical parameters represented in Table 1 and Figure 2. Excitation schematics of the piezo ceramic plates were also used, as shown in Figure 3. 

**Figure 14:**
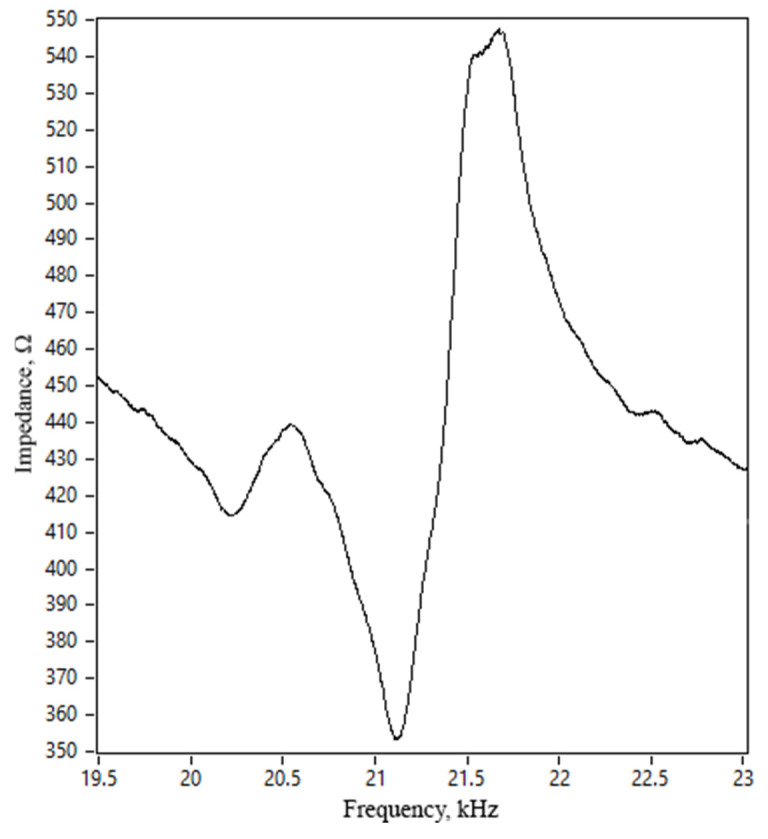
Impedance–frequency characteristics of motor.

Firstly, measurements of the impedance and phase frequency characteristics of the motor were recorded. A SinePhase 16777k (SinePhase, Mödling, Austria) impedance analyzer was used for this purpose. Figure 14 shows the measurement results.

We found that the resonance frequency of the motor was 21.18 kHz. The difference between calculated and measured resonance frequencies was 1.08 kHz or 4.87%. This error is mainly due to differences in material characteristics used for simulation and experiment, manufacturing, and assembling errors. Moreover, the measured impedance value was notably higher than the calculated frequency. This difference is due to the neglected glue layer in the numerical model and the reasons indicated earlier. The motor’s effective electromechanical coupling *k_eff_* was calculated with a value of 0.046. The difference between the calculated and measured values was 6.12%.

**Figure 15:**
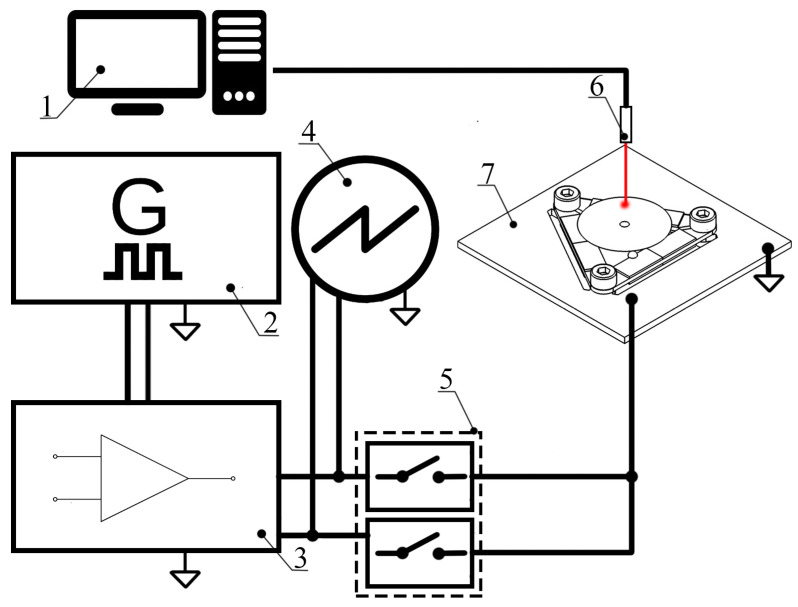
Schematics of experimental setup; 1—computer, 2—signal generator; 3—power amplifier; 4—oscilloscope; 5—signal junction box; 6—digital non-contact tachometer; 7—prototype of motor.

The next step of the experimental study was dedicated to recording the motor’s rotation speed measurements and torque. For this purpose, an experimental setup was built (Figure 15). 

The experimental setup included a computer, a signal generator WW5064 (Tabor Electronics, Nesher, Israel), a power amplifier PD200X4 (Piezo Drive, Shortland, Australia), an oscilloscope DL2000 (Yokogawa, Tokyo, Japan), a digital non-contact tachometer DT210 (Nidec-Shimpo, Nagaokakyo-City, Japan), and a custom-made signal junction box used to control the direction of rotation. The signal generator generated two sawtooth signals with a phase difference of π while the signal frequencies were the same and equal or close to the resonance frequency. Two signals were supplied to the signal junction box and used to implement switching between inducing clockwise and counterclockwise rotation of the rotor. An oscilloscope was used to control the amplitudes of the excitation signals. Rotation speed was measured using a digital non-contact tachometer that was connected to a computer and operated as a data acquisition device.

**Figure 16:**
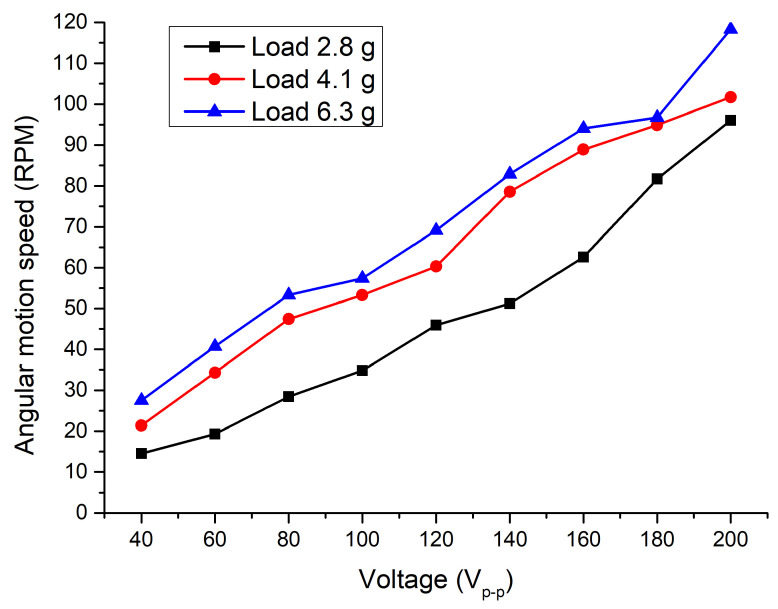
Dependance of angular speed from excitation voltage and load.

Figure 16 shows rotation speed measurements under three different preloads as the excitation voltage changed from 40 to 200 V_p-p_. The rotation speed increases in an almost linear trend while the voltage increases. The highest rotation speed was obtained at a voltage of 200 V_p-p_ while the preload was 6.8 g and reached 118.3 RPM or 0.59 RPM/V_p-p_. The lowest angular motion was obtained at 40 V_p-p_ while the preload was 2.8 g and reached 14.5 RPM or 0.36 RPM/V_p-p_.

**Figure 17:**
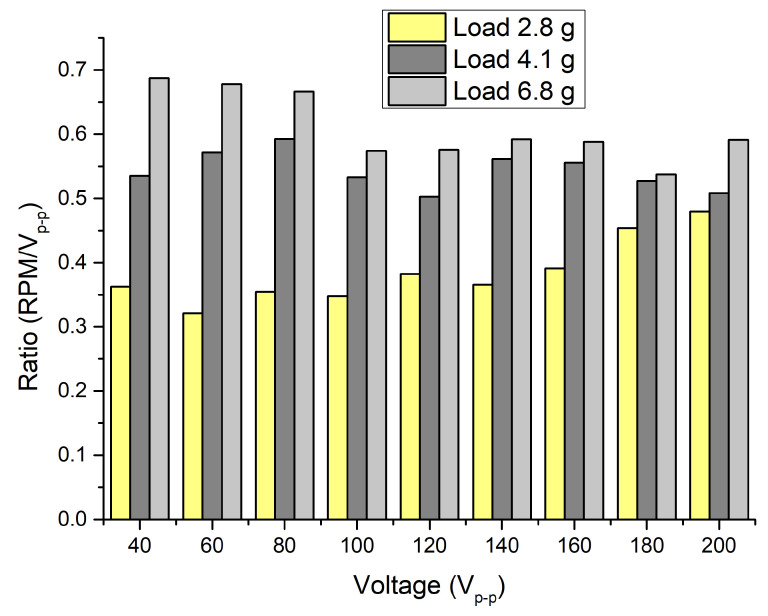
Ratio between the angular speed and excitation voltage at different loads.

Figure 17 shows a more accurate difference between rotation values under different preload and excitation voltage. The variation in ratios between rotation speeds and excitation voltage depends on preload, and the average speed per volt ratio is 0.38 RPM/V_p-p_ when the preload is 2.8 g. The deviation is 0.048. The average ratio at a preload of 4.1 g is 0.509 RPM/V_p-p_ and the deviation is 0.0279. Finally, the average ratio at a preload of 6.8 g is 0.61 RPM/V_p-p_ with a deviation of 0.0502. The lowest deviation was obtained at a preload of 4.1 g. Based on the obtained results, the proposed motor can operate under different electrical and mechanical load conditions, and its control can be easily and predictably implemented using closed- or open-loop control systems.

**Figure 18:**
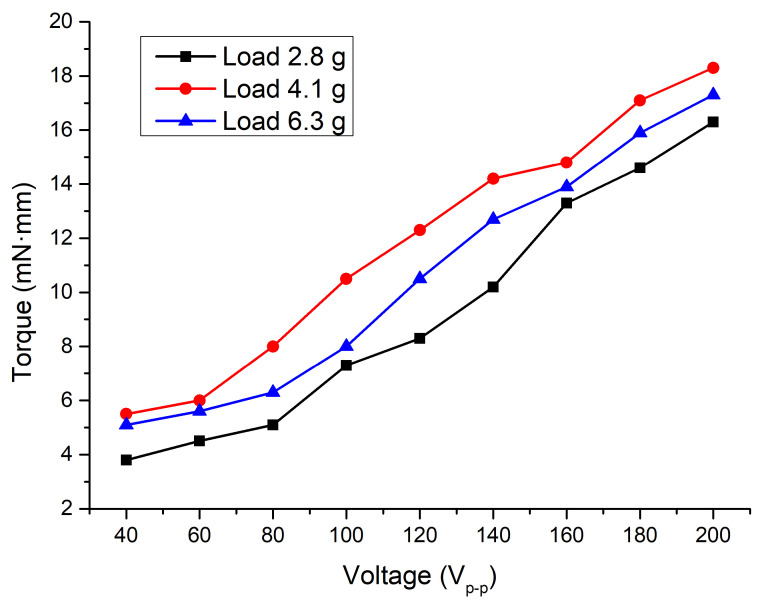
Dependence of torque on excitation voltage and load.

The next step of the experimental investigation was dedicated to torque measurements at different preload and voltage values. Figure 18 shows the measurement results.

The lowest torque values were obtained when a preload of 2.8 g was applied to the motor. It reached 3.8 and 16.3 mN·mm when voltages of 40 and 200 V_p-p_ were applied, respectively. On the other hand, the lowest torques had values of 5.5 and 5.1 mN·mm at preloads of 4.1 and 6.3 g. The highest torque of 18.3 mN·mm was obtained when a voltage of 200 V_p-p_ and preload of 4.1 g were applied. Moreover, deviating torque values via a whole range of excitation signal amplitudes at 2.8, 4.1, and 6.3 g were 4.34, 4.26, and 4.48, respectively. Therefore, these values and their deviations show that the motor can provide stable output torques at different loads and voltages. Therefore, it can be found that the motor is able provide suitable angular motion speed at different excitation voltage levels and preload while operation of motor is represented in Video S1 of supplementary materials.

Table 3 compares the proposed motor’s mechanical output characteristics and small-size traveling wave piezoelectric motors. The rotary motors were compared by comparing the ratio between performance and weight because direct comparison is complicated due to differences in operation frequencies, preload forces, etc.

The proposed motor has a higher ratio of angular speed to weight although the excitation signal amplitudes of other motors are higher. On the other hand, the ratio of output torque to weight is lower than other motors. However, considering that the weight of traveling wave motors is several times higher, the output torque ratio to the weight of the proposed motor is similar to that of the compared motors.

## 5. Conclusions

In the present study, a novel low-profile piezoelectric rotary motor comprising three bimorph piezoelectric plates was introduced and thoroughly examined. The optimal positioning of spherical contacts was determined, revealing that the ratio between displacement amplitudes in the Y and Z directions had increased sixfold. Additionally, time domain study results confirmed the operation principle of the motor.

Experimental validations of the motor prototype were conducted to assess its performance. The measurements confirmed an operational frequency of 21.18 kHz. Furthermore, experimental investigations demonstrated the motor’s ability to achieve rotation speeds of up to 118 RPM and generate torque of up to 18.3 mN·mm. Notably, experimental studies revealed the rotation speed’s almost linear dependence on the applied voltage, emphasizing the motor’s controllable and predictable behavior.

## Figures and Tables

**Table 1:** Geometrical parameters of stator.

Parameter	Value	Description
L	40 mm	Total length of stator
L_plate_	26.65 mm	Length of piezoelectric bimorph plate
L_PZT_	10 mm	Length or piezo ceramic plate
L_pos_	5 mm	Longitudinal position of spherical contact
R_fix_	1.6 mm	Radius of cavity for stator clamping
R_contact_	1 mm	Radius of spherical contact
W_cut_	1 mm	Width of decoupling cut
W_PZT_	5 mm	Width of piezo ceramic plate
t	2 mm	Thickness of stator
t_PZT_	0.5 mm	Thickness of piezo ceramic plate

**Table 2:** Material properties used in the model.

Material Properties	Stainless Steel DIN 1.4301	CTS Corp NCE81	Aluminum Oxide Ceramic
Density, [kg/m^3^]	8000	7730	3980
Young’s modulus, [N/m^2^]	193 × 10^9^	-	41.9 × 10^10^
Poisson’s coefficient	0.29	0.32	0.33
Isotropic structural loss factor	0.02	-	0.2 × 10^−3^
Relative permittivity	-	ε_33_^T^/ε_0_ = 1020	-
Elastic compliance coefficient [10^−12^ m^2^/N]	-	S_11_^E^ = 16.00 S_33_^E^ = 17.00	-
Elastic stiffness coefficient c_33_^D^, [N/m^2^]	-	16.6 × 10^10^	-
Piezoelectric constant d_33_ [10^−12^ m/V]	-	255	-
Piezoelectric constant d_31_ [10^−12^ m/V]	-	−100	-

**Table 3:** Comparison between piezoelectric motors.

Reference	Motor Operation Principle	Footprint Area	Weight	Angular Speed/Torque	Ratio of Output Speed/Torque to Motor Weight
Introduced motor	Inertial principle	415 mm^2^	4.2 g	Torque 18.3 mN·mm @ 200 V_p-p_ Angular speed 118 RPM @ 200 V_p-p_	Ratio of torque/weight = 4.36 mN·mm/g Ratio of angular speed/weight = 28.09 RPM/g
Ma et al. [25]	Traveling wave	2826 mm^2^	30 g	Torque 0.11 N·m @ 250 V_p-p_ Angular speed 33.36 RPM @ 250 V_p-p_	Ratio of torque/weight = 3.66 mN·m/g Ratio of angular speed/weight = 1.112 RPM/g
Ren et al. [26]	Model—PMR45 —traveling wave motor	2551 mm^2^	143.3 g	Torque 0.4 N·m @ 400 V_p-p_ Angular speed—245 RPM @ 400 V_p-p_	Ratio of torque/weight = 2.7 mN·m/g Ratio of angular speed/weight = 1.71 RPM/g

## Data Availability

Dataset available on request from the authors.

## Supplementary Materials

The following supporting information can be downloaded at: https://www.mdpi.com/article/10.3390/mi15010132/s1; Video S1: Operation of motor.
